# Preparation of orodispersible tablets of bosentan using xylitol and menthol as dissolution enhancers

**DOI:** 10.1038/s41598-024-60494-9

**Published:** 2024-05-09

**Authors:** Rania Mohamed Sakr, Abdelaziz El Sayed Abdelaziz, Eman Ahmed Mazyed, Gamal Mohamed El Maghraby

**Affiliations:** 1 Department of Pharmaceutical Technology, Faculty of Pharmacy, Kafrelsheikh University, Kafr El Sheikh, Egypt; 2 Department of Pharmaceutical Technology, Faculty of Pharmacy, Tanta University, Tanta, Egypt

**Keywords:** Drug development, Drug discovery

## Abstract

Bosentan is a drug used to treat pulmonary hypertension via dual endothelial receptor antagonism. Bosentan has a restricted oral bioavailability, a problem that's mostly due to poor solubility and hepatic metabolism. It is extensively used for the elderly and children who require a friendly dosage form like orodispersible tablets. So, the goal of this research work was to hasten the dissolution rate of bosentan to produce an orodispersible tablet with immediate drug release. Bosentan was exposed to ethanol-assisted kneading with a rise of xylitol or menthol concentrations (1:1 and 1:2 molar ratio of bosentan with excipient). In addition to observing the dissolution behavior, the resulting dry products were investigated using Fourier transform infrared spectroscopy (FTIR), differential thermal analysis (DTA), and X-ray diffraction (XRD). The FTIR reflected possible hydrogen bonding with xylitol and menthol. DSC studies reflected a reduction in the enthalpy and Tm. These results with XRD data reflected partial co-amorphization in the case of xylitol and eutaxia in the case of menthol. These modifications were related to an accelerated dissolving rate. The developed systems were fabricated as orodispersible tablets which exhibited immediate release of bosentan. Thus, the current study offered simple co-processing for the preparation of orodispersible bosentan tablets.

## Introduction

Bosentan hydrate is a sulfonamide-based compound that was approved for the therapy of pulmonary arterial hypertension^1^. The use of bosentan was extended to other diseases including Eisenmenger syndrome, persistent pulmonary hypertension of the newborn, etc.^2,3^. Bosentan is a weakly acidic, highly lipophilic drug (log *P* = 4.94), and melts at 104 °C. These parameters offered bosentan high membrane permeability and low water solubility (less than 2 μg/mL). It was thus included in the BCS class II drug list^4–6^. The biopharmaceutical and pharmacokinetic specifications highlighted the availability of bosentan in two strengths (62.5 mg and 125 mg tablets). The studies also revealed problems associated with traditional tablets including the need for frequent dosing and low oral bioavailability (50%) which minimized the therapeutic benefits^6–9^. The drug is also subject to hepatic metabolism which can contribute to poor bioavailability^10–13^. Considering these characteristics with the target patients of this drug which can include extreme age for both children and elderly, the development of oral disintegrating tablets can provide an advantage for the drug and these patients^14,15^. Unfortunately, this development is hampered by poor drug dissolution which requires improvement.

Literature reports presented some attempts to improve the dissolution characteristics of bosentan. These investigations utilized solid dispersion techniques using Gelucire® and Poloxamer® as low melting point surfactants^16,17^. Other solid dispersions utilized hydroxy propyl β-cyclodextrin (HPβ-CD) and polyethylene glycol (PEG-4000) as inert hydrophilic polymers^18^. Self-nanoemulsifying drug delivery system (SNEDDS) was also utilized for this purpose^4,6,19^. Other authors prepared microemulsions using rice bran, sunflower, or Capmul® as the oily phase with suitable surfactant/cosurfactant systems^20,21^. Co-spray drying with mannitol was also presented as a tool for dissolution enhancement of bosentan^22^. Other teams adopted inclusion complexation with cyclodextrin and utilized novel hybridization techniques^23,24^. The sophistication was extended to prepare nanosuspensions which showed dissolution enhancement^2^.

Alteration of the crystalline structure by simple co-treatment with hydrophilic excipients can provide a promising alternative with high potential for scaling up. Modification of the crystalline structure can be in the form of amorphization, eutectic mixture formation, or co-crystallization. The dissolution characteristics of many medications have been improved because of these alterations^25–31^.

The aim of this study was to determine how ethanol-assisted kneading of bosentan with menthol or xylitol modified the rate of the dissolution of bosentan. The purpose was expanded to design and test orodispersible tablets with immediate drug release. Xylitol and menthol were utilized for the alteration of the crystalline structure for the dissolution enhancement. Their benefits are widened based on their flavoring effect in orodispersible tablets in addition to the possible membrane permeability enhancing effect of menthol^26,31–35^

## Materials and methods

### Materials

Bosentan monohydrate was gifted as a free sample from Eva Pharma for Pharmaceutical and Medical Appliances, Cairo, Egypt. Menthol was supplied from Adwic Pharmaceutical Chemicals Company, Cairo, Egypt. Xylitol, Avicel®, and magnesium stearate were provided by Sigma for Pharmaceutical Industries, Qwesna, Egypt. Crospovidone sodium, Croscarmellose sodium were provided as gift samples from Utopia, Badr city, Egypt. Pharmaceutical-grade ethyl alcohol was procured from Lanxess, Dortmund, Germany. Sodium lauryl sulfate was purchased from DOP Organik Kimya, Ankara, Turkey*.*

### Preparation of formulations

Mixtures containing bosentan with menthol or xylitol were prepared by ethanol-assisted kneading based on the molar and weight ratios shown in Table 1^36^. Briefly, bosentan was subjected to dry mixing with menthol or xylitol before a gradual addition of ethanol with continuous mixing to provide a smooth paste. Avicel® was added to the paste and mixing continued to prepare dry powder. This mixture was preserved in an airtight container until it was needed. Bosentan powder was subjected to ethanol-aided kneading in the absence of any additives to prepare the positive control. Physical mixtures containing bosentan with either xylitol or menthol at 1:2 molar ratios were prepared by gentle mixing of the dry powders with a spatula.

**Table 1:** The compositions of the tested formulations expressed as molar and weight ratios.

Formulation	Bosentan	Menthol	Xylitol	Avicel®
Pure drug	1	0	0	0
Positive control	1	0	0	0
X1	1 (1)	0 (0)	1 (0.267)	1 (1)
X2	1 (1)	0 (0)	2 (0.534)	1 (1)
M1	1 (1)	1 (0.27)	0 (0)	3 (3)
M2	1 (1)	2 (0.55)	0 (0)	3(3)

### FTIR spectroscopy

The effect of ethanol-assisted kneading with menthol or xylitol was assessed using FTIR. Thus, the FTIR spectral features of pure bosentan, xylitol, menthol, and formulations containing bosentan with xylitol or menthol at 1:1 or 1:2 molar ratios were researched. This was performed using FTIR equipment (PerkinElmer, model Spectrum one, Waltham, MA). Sample preparation involved compression of the test material into circular disks with potassium bromide. The compressed disk was mounted on the sample holder and scanned with FTIR spectrophotometer within the range of 4000–400 cm^−1^. The gathering and management of the data were attained by OMNIC software coupled with a deuterated triglycine sulfate detector DTGS detector**.**

### Differential scanning calorimetry (DSC)

Thermal analysis was performed on pure bosentan, xylitol, menthol, and the prepared formulations using DSC equipment (DTA) (Discovery DSC 25-TA instrument, Newcastle, DE, USA). The sample (2–4 mg) was loaded in a sample pan made of Aluminum. The lid was mounted, and the pan was sealed by compression. This was held on the furnace with an empty pan being mounted as a reference. Each material's thermogram was captured at temperatures range 30 to 400 °C with an increment rate being adjusted to 10 °C/min. Heating was done with a constant stream of nitrogen gas (50 ml/min). Results capture and manipulation were attained using TRIOS software.

### Powder X-ray diffraction (PXRD)

The effect of kneading with menthol or xylitol on the crystallinity of bosentan was evaluated by PXRD. The crystalline structure of the bosentan, xylitol, menthol, and the developed formulation was evaluated via PANalytical (Almelo, Netherlands). This equipment is supported with a point detector (gas-filled detector) Diffractograms were recorded in the 2θ range of 6–60° with a scanning step size of 0.03°.

### Spectroscopic analysis of bosentan monohydrate and method validation

A stock solution containing 1000 µg/ml of bosentan monohydrate was prepared in ethanol. This was utilized to produce a set of concentrations (6, 8, 10, 14, 18, and 20 µg/ml) by suitable dilution with the dissolution medium (1% sodium lauryl sulfate in water). The absorbance values of these concentrations were measured at 240 nm using a blank containing the same solvent composition. The equipment used was a T80 + UV/Vis double beam spectrophotometer, PG Instruments, Ltd (Leicestershire, United Kingdom). Plotting the absorbance readings against the corresponding concentrations led to the creation of the standard graph. The graph with fitted to a straight line and the equation was used for the quantification of unknown samples.

### Characterization of powder blends

Carr index, Hausner's ratio, and angle of repose measurements were made to track the flow behavior of the powder blends. Briefly, the bulk density (ρb) was estimated from the bulk volume which was obtained by pouring an estimated weight of the powdered mixture into a measuring cylinder followed by manual gentle tapping (3 times) to level the surface. ρb was computed as powder weight per bulk volume. The tapped volume was estimated by manual tapping of a graduated measuring cylinder containing a powder sample 100 times. The tapped density (ρt) was then computed as the weight per the tapped volume^28^. Carr index (compressibility index) and Hausner’s ratio were calculated using the following equations.

$$ {\text{Carr Index }}\left( \% \right) \, = \, \left[ {\left( {\rho {\text{t }}{-} \, \rho {\text{b}}} \right)/ \, \rho {\text{t}}} \right] \, \times { 1}00 $$

$$ Hausner^{\prime}s \, ratio \, = \, \rho t \, / \, \rho b $$

The angle of repose (α) was also estimated by pouring the powdered mixture through a vertically positioned funnel at a fixed height (h). Pouring continued to form a heap having the predetermined height and the radius of this heap (r) was measured to determine α using the following equation:

$$ {\text{tan}}\,\alpha = {\text{ h }}/{\text{ r}} $$

### Dissolution studies

Using the USP II dissolution technique, the dissolution of bosentan was evaluated before and after processing. The paddle revolved at 50 rpm while the dissolution fluid (1% w/v sodium lauryl sulfate in distilled water) was applied. The dissolution medium and paddle speed were selected based on previous literature^17^. The dissolution equipment was LSCI dissolution test model ITDD-06 Languedoc Scientifique, Rivesaltes, France. Sodium lauryl sulfate (1% w/v, 900 ml) was added into the vessels and was adjusted to 37 ± 0.5 °C before loading 125 mg of bosentan in the form of pure drug, its equivalent formulation, or physical mixture onto the vessels. Collection of samples (5 ml) was done 5, 10, 15, 30, 45, and 60 min after loading followed by immediate filtration using a 0.45 µ cellulose filter. Fresh medium corresponding to each sample was immediately added after each sampling to keep the constant volume. Each sample's drug content was determined using the UV spectroscopic technique after dilution (1:1) with ethanol to avoid any interference from air bubbles generated from sodium lauryl sulfate. Bosentan's cumulative dissolved concentration was represented as a percentage of the dose before plotting against the sampling time points. This generates the dissolution profiles that were employed to determine the dissolution parameters including Q5 (% dissolved within the first 5 min), Q10 (% dissolved after 10 min), Q60 (% dissolved after 60 min), and dissolution efficiency (DE %). The latter was calculated from the area under the dissolution profile of a specific formulation relative to the comparable region assuming 100% dissolution at all time points^37^. For statistical comparison between formulations, the computed dissolving parameters were used. In addition, dissolution profiles were compared using the similarity factor test which was computed using the proceeding equation:

$$F2=50\times log\{[1+\frac{1}{n}\sum_{t-1}^{n}{(Rt-Tt)]}^{-0.5} x 100$$

where n denotes for the number of data points used in the calculation, Rt is the percentage of bosentan released from the first formulation at time t and Tt is the percentage of bosentan released from the second formulation at the same time^28,38^.

### Preparation of fast disintegrating tablet

The bosentan formulations undergoing fast release of the drug were adopted in fabricating fast disintegrating tablets with subsequent rapid dissolution. The tablets were formulated according to the composition presented in Table 2. Bosentan was subjected to wet kneading with menthol or xylitol to prepare the dry flowable powder as mentioned above. The prepared formulation was combined geometrically with the remaining tablet excipients before compression into tablets with the pressure adjusted to produce tablet hardness of around 5 kilo pounds (kp). This utilized a 14 mm punch of single punch tablet machine (Royal Artist, Kapadia Industrial Estate, BLDG, Mumbai, India).

**Table 2:** The composition of fast disintegrating tablets.

Ingredients	X2T (xylitol)	M2T (menthol)
Bosentan (mg)	125	125
Menthol (mg)	0	68.58
Xylitol (mg)	66.78	0
Crospovidone sodium (mg)	30	30
Croscarmellose sodium (mg)	30	30
magnesium stearate (mg)	6	6
Avicel® to (mg)	600	635

Bosentan was subjected to kneading with menthol or xylitol in the presence of Avicel®.

### Evaluation of fast-disintegrating tablets

Quality control tests were performed on fast-disintegrating tablets**.** The weight uniformity was estimated by recording the individual weight of 20 tablets and computing the deviation of the weight of each tablet from the mean weight of the selected tablets. This deviation was expressed as a percentage and the tablets were considered acceptable if no more than two tablets differed by more than 5% with no tablet deviating by more than double the limit^36^**.**

Tablet friability was measured using an Erweka friability tester (Heusenstamm, Hesse, Germany). Before subjecting ten tablets to 100 revolutions, their initial weight was measured. The friability was calculated from the difference between the starting and final weights represented as a percentage of the initial weight for intact tablets after dedusting and weighing. Acceptance was indicated if the friability was not more than 1%^39^.

The uniformity of content was determined by selecting 30 tablets at random, A drug content analysis was performed on 10 of them. This entailed powdering the tablet before dissolving it in ethanol with the aid of sonication. The dispersion was clarified by filtration and bosentan content was determined spectrophotometrically after suitable dilution. The acceptance was indicated if the individual contained 85–115% of the labeled bosentan. If the limit is exceeded by more than one tablet, the contents of all 20 remaining tablets must be quantified, and acceptance is justified if every single one of them falls inside the limit. Rejection was indicated if the content of any tablet was outside the range of 75% to 125% of bosentan^28^**.**

The disintegration test employed Copley Scientific apparatus (Model: NE4-COP, UK). This is supported by a basket unit that can hold 6 tablets. The loaded assembly moves up and down into 1 L of distilled water equilibrated to 37 ± 0.5 °C. Disintegration time was calculated after the complete fracture of tablets and the escaping of the particles outside the screen of the basket.

The wetting time was determined after loading the tablets on the surface of a wet filter paper placed in a Petri dish. The tablet surface was covered with Allura red crystals, and the time required for the surface to turn red was used as the wetting time. The test was done four times, and the average and standard deviation were computed.^28^.

Using an Erweka hardness tester (Heusenstamm, Hesse, Germany), the tablet's hardness was determined. The average hardness of 6 tablets was computed.

Additionally, using a calibrated caliper, the compressed tablets' thickness and diameter were measured.

The dissolution behavior of bosentan was monitored after tableting. This was achieved using the same technique as mentioned above in which the intact tablet replaced the powdered formulation. Immediate release marketed bosentan tablets (Bosentadin 125 mg/tablet, Eva Pharma, Egypt) were tested and were used for comparison.

### Stability studies

The disintegration time of the tablet and the release behavior of bosentan were tracked during the stability investigation after on shelf storage in an airtight dark brown glass container at room temperature (25 °C ± 2 °C/65% ± 5% RH). At predefined time intervals, the stored tablets were sampled before the determination of the disintegration time and release pattern. The selected test parameters are considered key factors contributing to the success of rapidly disintegrating tablets.

### Statistical analysis

To estimate the difference between formulations, the statistical study used the Kruskal–Wallis test, with Tukey's multiple comparison used as a post hoc test.

## Results

### FTIR spectroscopy

**Figure 1:**
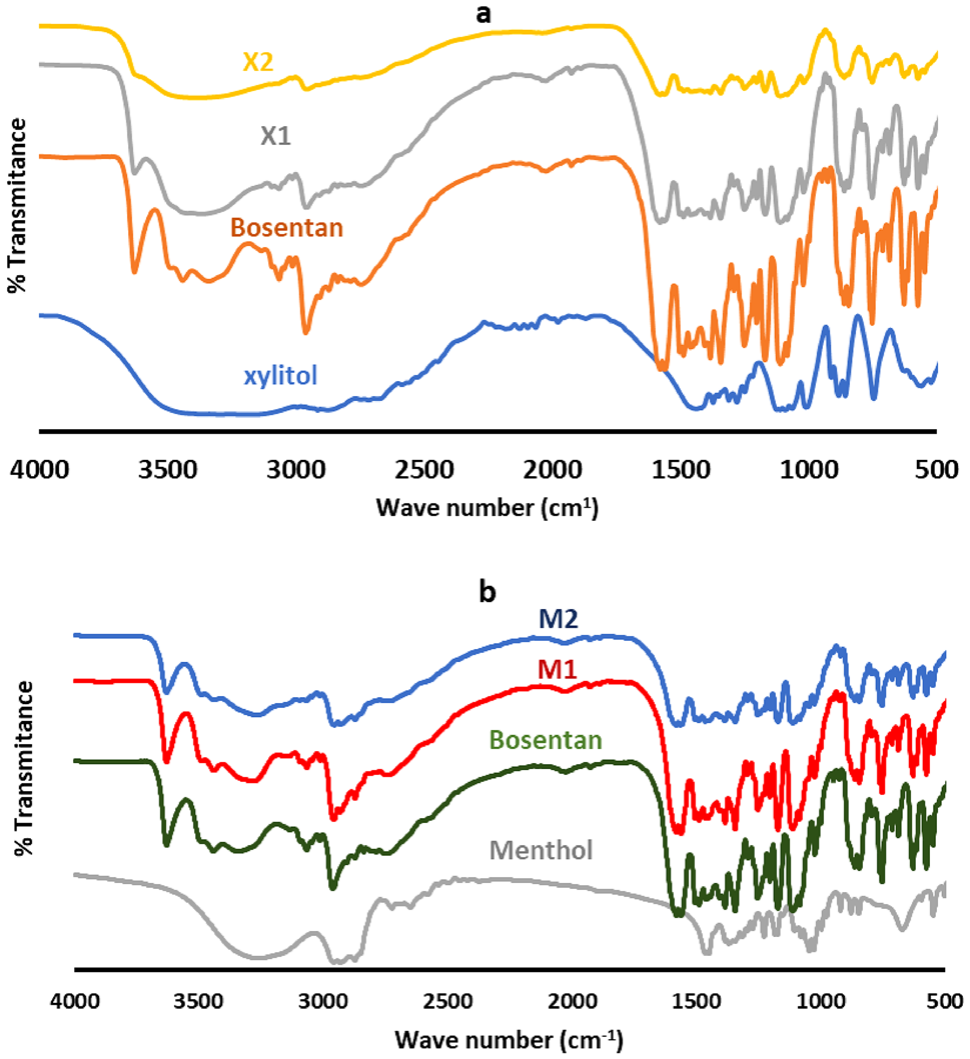
FTIR spectra of pure bosentan, xylitol, menthol, and their co-processed mixtures (X1, X2, M1 and M2). Formulation details are in Table 1.

Figure 1 shows the FTIR spectrum for bosentan, xylitol, menthol, and their co-processed formulations. The FTIR spectrum of pure bosentan is distinguished by absorption bands that correspond to the compound's functional groups. These bands included 3635 cm^−1^ for OH stretching, 3459 for NH stretching, 2960 cm^−1^ for CH-stretching of the aromatic ring, and 1578 and 1591 cm^−1^ for C=C stretching vibrations. The C–N stretching appeared at 1170 cm^−1^ with the CO stretching at 1257 cm^−1^. The spectrum also displayed the structural properties of SO2 as an absorption band at 1348 cm^−1^ for asymmetric stretching vibrations with SO2 symmetric stretching vibrations appearing as two bands at 1121 and 1026 cm^−1^. The in plane bending of the hydroxyl group was evident at 1389 cm^−1^. The wagging of NH was noticed at 753 cm^−1^ (Fig. 1).

The FTIR spectrum of xylitol expressed a very broad absorption band in the range of 3645–2800 cm^−1^ for the hydrogen bonded OH groups. The out of plane (OOP) bending of OH was noticed as an intense absorption band at 759 cm^−1^ with the in-plane bending showing at 1446 cm^−1^. The CH2 rocking of the xylitol backbone appeared in the range of (918 to 860 cm^−1^). The absorption bands at 1284, 1270, and 1016 cm^-1^ denote the C–O stretching vibrations (Fig. 1).

The FTIR spectrum of menthol showed a broad absorption band for the OH stretching at 3300 cm^−1^. The CH stretching vibrations appeared at 2890 and 2970 cm^−1^. The OOP and in-plane bending of OH appeared at 688 cm^−1^ and 1230 cm^−1^, respectively (Fig. 1).

Wet co-grinding of bosentan with xylitol at 1:1 or 1:2 molar ratios yielded a product showing a broadening of the absorption band of the NH group of bosentan to become flat in the mixture containing bosentan with xylitol at a 1:2 molar ratio. This was associated with broadening and shifting to a lower wave number of the bands corresponding to the CN stretching. The change in the absorption band of NH wagging was not clear due to the combination with an absorption band corresponding to the OH bending of xylitol. Moreover, the asymmetric SO2 stretching band was broader. The absorption bands corresponding to the CO stretching of xylitol were broader with a shift to a lower wave number.

Similarly, the co-processing of bosentan and menthol at 1:1 or 1:2 molar ratios broadened the absorption bands corresponding to the CN stretching and NH bending (Fig. 1).

### Differential scanning calorimetry (DSC)

**Figure 2:**
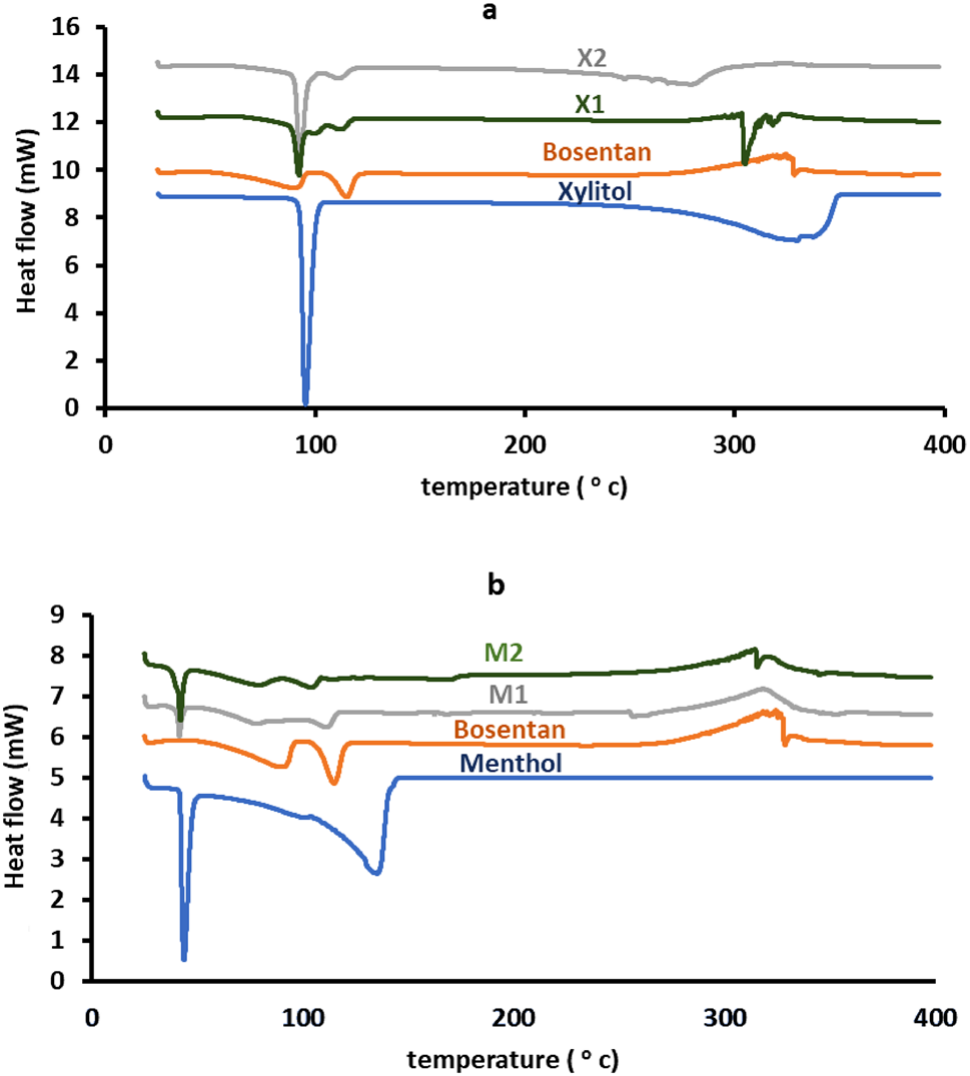
Representative DTA thermograms of pure bosentan, xylitol, menthol, and their co-processed mixtures (X1, X2, M1 and M2). Formulation details are in Table 1.

Figure 2 shows examples of the recorded DSC thermograms of bosentan before and after ethanol-aided kneading with menthol or xylitol. These thermograms were used to determine the thermal characteristics which are listed in Table 3. The raw material of bosentan exhibited thermal behavior reflecting two endotherm peaks at 87.2 °C and 112.24 °C. The thermogram also showed an exothermic peak at 323 °C.

**Table 3:** The thermodynamic parameters of pure bosentan, menthol, xylitol, and different formulations.

Formulation	Onset (^O^C)	Endset (^O^C)	Tm (^O^C)	Enthalpy J/g
Unprocessed drug	62.6	94	87.2	77.23
	102	121.7	112.24	46.35
Xylitol	86	101.9	94.8	230
Menthol	35.7	48.9	43.71	87.34
X1	84.2	94.2	91.4	247
	102.7	121.7	109	14
X2	83.1	102	92	250
	102	112.5	108	21.5
M1	32.26	43.1	41.2	46.45
	102	115	106.8	13.49
M2	32.26	44.2	41.4	65.59
	89	105.5	101	18.8

Raw xylitol showed a thermal pattern exposing two endothermic transitions, The first was observed as a sharp peak at 94.8 °C and the second was recorded as a broad peak at 327 oC (Fig. 2 and Table 3). The thermal pattern of menthol was characterized by sharp and broad endothermic peaks that appeared at Tm values of 43.7 °C and 172.8 °C, respectively.

Ethanol influenced kneading of bosentan with xylitol produced a solid product with thermal behavior showing modulations in the enthalpy of the transition of the drug to be reduced from 46.35 J/g to reach 14 and 21.5 J/g after kneading with xylitol at weight ratios of 1:1 and 1:2, respectively. The reduction in enthalpy was associated with a decrease in the endset and Tm of melting transition. The thermogram of the formulations showed the melting transition of xylitol (Table 3).

Kneading bosentan with menthol produced a dry product with altered thermograms in comparison with pure bosentan. The alterations were shown as significant changes in the Tm of the melting transition of bosentan. The Tm decreased to 106.8 and 101 °C after kneading bosentan with menthol at 1:1 and 1:2, molar ratios, respectively. The modulation in Tm was linked with a decrease in the enthalpy (Fig. 2 and Table 3).

### Powder X-ray diffraction (PXRD)

**Figure 3:**
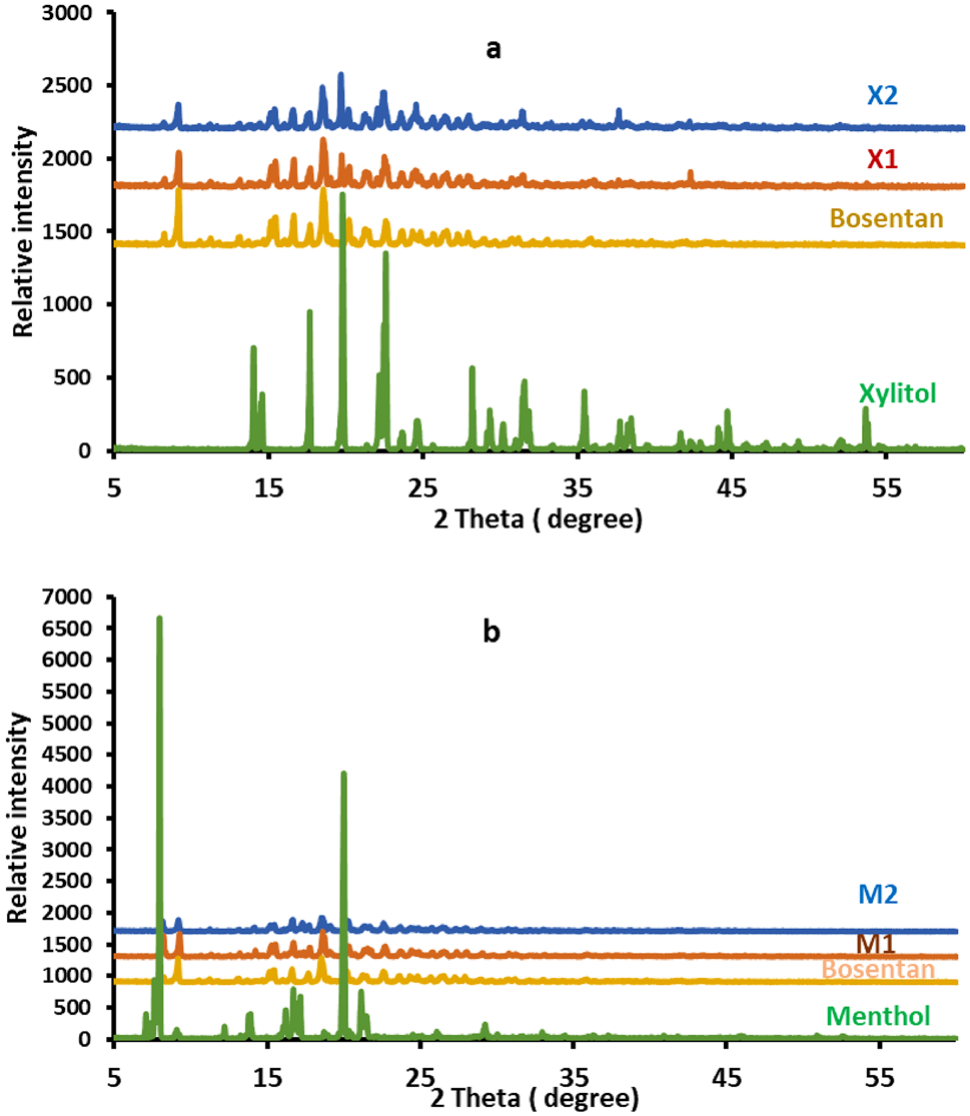
X-ray diffraction pattern of pure bosentan, xylitol, menthol, and their co-processed mixtures (X1, X2, M1 and M2). Formulation details are in Table 1.

Figure 3 displays the patterns of X-ray diffraction of pure bosentan, xylitol, menthol, and their co-processed formulations, the diffraction peaks are also displayed. The raw bosentan diffractogram demonstrated intense diffraction peaks available at 2θ values of 8.29, 9.33, 10.55, 11.25, 13.17, 15.45, 16.63, 17.71, 18.75, 20.19, 21.45, 22.65, 23.55, 24.33, 24.83, 25.73, 26.65, 27.37 and 27.9 (Fig. 3).

The diffraction peaks of xylitol were shown at 2θ values of (14.05, 14.45, 17.67, 19.85, 22.19, 22.61, 23.71, 24.69, 28.17, 29.33, 30.2, 31.5, 35.43, 37.75, 38.45, 44.19, 44.7 and 53.65). The diffractogram of the pure menthol showed diffraction peaks at 2θ values of 7.93, 9.25, 12.19, 13.93, 16.2, 17.17, 19.95, 21.15 and 29.21 (Fig. 3).

kneading of bosentan with xylitol produced a crystalline product with a diffractogram demonstrating diffraction peaks that are broader and less intense than those of the parent compound, bosentan (Fig. 3).

Kneading with menthol produced solid crystals with an XRD pattern in which the position of the diffraction peaks was preserved but appeared with lower intensity and showed broadening compared to the raw material (Fig. 3).

### Pre-compression characteristics of powdered mixture

Table 4 presents the characteristics of the powdered mixtures of tablet formulation. The computed values of the Carr index, Hausner`s ratio, and angle of repose collectively indicate acceptable flow behavior.

**Table 4:** The pre-compression characteristics of powder mixtures.

Parameter	X2T (xylitol)	M2T (menthol)
Bulk density of the powder mixture (g/cm^3^)	0.27	0.229
Tapped density of the powder mixture (g/cm^3^)	0.32	0.277
Hausner’s ratio	1.18	1.20
Carr index (%)	15.60	17.32
Angle of repose (α) (^o^)	27.70	25.89

### Dissolution studies

**Figure 4:**
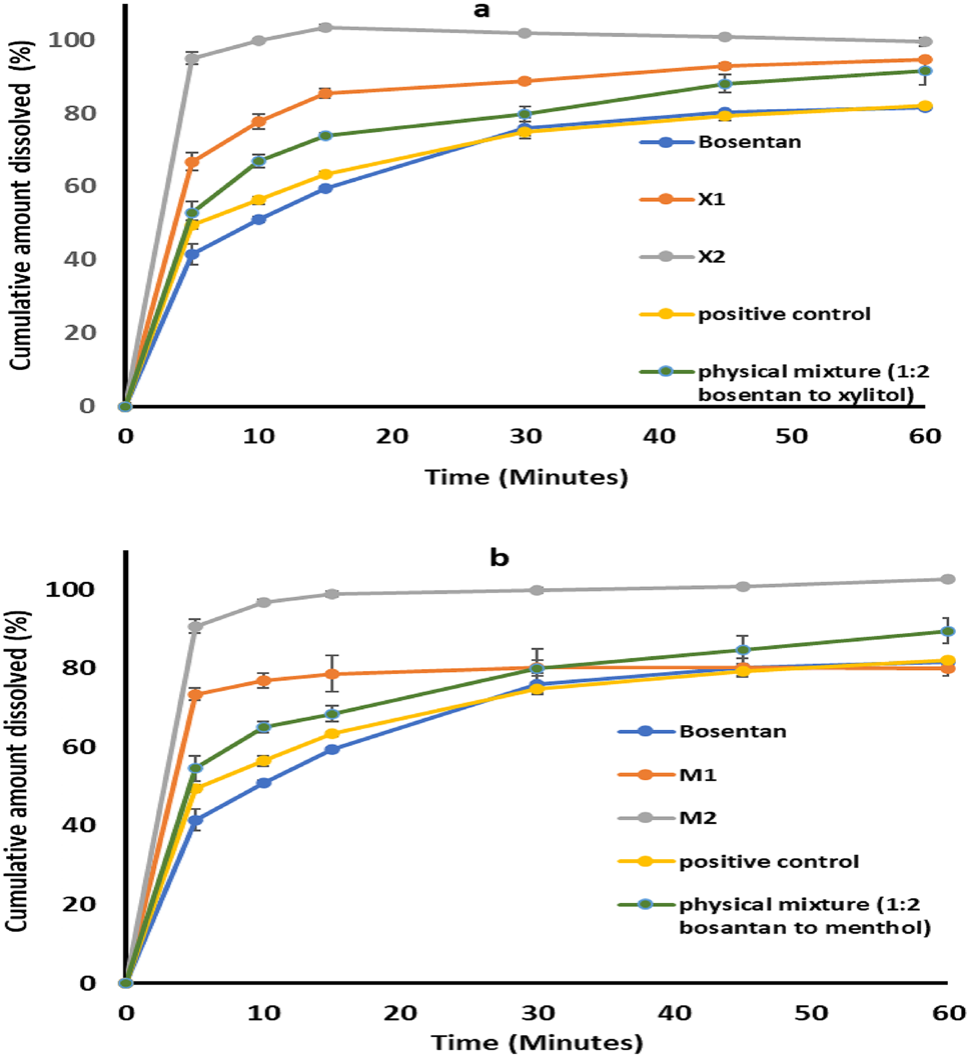
In vitro dissolution of bosentan from its unprocessed powder or its co-processed mixtures with xylitol (A) or menthol (B). Formulation details are in Table 1.

Figure 4 represents the dissolution profiles, and Table 5 lists the computed dissolution parameters. The unprocessed bosentan showed slow release was seen as small Q5 values (41.47%) with a total dissolution efficiency of 66.86%. Kneading of bosentan with ethanol in the absence of any additives followed by drying produced solid material with dissolution parameters in which the Q5 value was 49.46% and the total dissolution efficiency 68.15% (Fig. 4 and Table 5).

**Table 5:** The dissolution efficiency, Q5, Q10, and Q60 values of pure unprocessed bosentan, wet ground drug, and the prepared formulations.

Formulation	Q5 (%)	Q10 (%)	Q60 (%)	DE (%)
Pure drug	41.47 ± 2.74	50.97 ± 0.70	81.63 ± 0.60	66.86 ± 1.16
Positive control	49.46 ± 0.96	56.48 ± 1.31	82.03 ± 0.57	68.15 ± 0.23
Physical mixture (1:2, bosentan to xylitol)	52.85 ± 3.15	66.90 ± 1.69	91.51 ± 3.60	75.73 ± 1.30
Physical mixture (1:2, bosentan to menthol)	54.6 ± 3.18	65.14 ± 1.4	89.49 ± 3.14	73.76 ± 2.06
X1	66.8 ± 2.32	77.70 ± 1.99	94.7 ± 0.45	83.55 ± 0.81
X2	95.08 ± 1.59	99 ± 0.59	99.5 ± 1.071	96.62 ± 0.27
M1	73.4 ± 1.48	76.81 ± 1.92	80 ± 1.82	75.71 ± 3.80
M2	90.6 ± 1.77	96.71 ± 0.65	102.6 ± 0.66	95.07 ± 0.23

Ethanol-aided kneading of bosentan with xylitol produced an appreciable rise in the dissolution rate of bosentan as reflected by the considerable improvement in dissolution parameters in comparison to the unprocessed or processed bosentan (*P* < 0.05). The magnitude of dissolution enhancement depended on the ratios of bosentan to xylitol. Thus, in the first 5 min, a formulation including bosentan and xylitol at a 1:1 molar ratio released 66.8% of the bosentan, and the recorded total dissolution efficiency was 83.55%. Doubling the ratio of xylitol increased the dissolution parameters to release 95.08% during the first five minutes with a total dissolution efficiency of 96.62%. Statistical analysis of the difference between the dissolution parameters indicated the superiority of formulation containing a higher proportion of xylitol compared to that containing a 1:1 molar ratio (*P* < 0.05). The similarity factor test, in which the F2 value was 28%, provided additional evidence of its superiority. The dissolution of bosentan from a physical mixture containing xylitol (1:2, molar ratio) was greater than the unprocessed drug to record Q5 of 52.85%, Q10 of 66.9%, and a dissolution efficiency reaching 75.73%. However, these values remained significantly lower (*P* < 0.05) than that recovered from the corresponding formulation (Fig. 4 and Table 5).

Ethanol-enhanced kneading of bosentan with menthol showed a major enhancement in bosentan dissolution as noticed from the calculated dissolution parameters (P < 0.05). The augmentation depended on the ratios of bosentan to menthol with the formulation containing drug and menthol at a 1:2 molar ratio providing the fastest dissolution rate. Thus, the Q5 values were 73.4% and 90.6% for formulations containing drug and menthol at 1:1 (M1) and 1:2 (M2) molar ratios, respectively. For the same formulae, the values of the overall dissolving efficiency were 75.7% and 95.07%, respectively. The difference between both formulations was statistically significant. Considering the overall dissolution profile, the similarity factor test elected the M2 formulation to be better than M1 (F2 = 38%). The physical mixture of bosentan with menthol (1:2, molar ratio) dissolved in higher proportions compared with the pure bosentan. Q5 was 54.6%, Q10 was 65.14% and dissolution efficiency reached 73.76%. Again, these values remained significantly lower (*P* < 0.05) than those recovered from the corresponding formulation.

### Characterization of fast-disintegrating tablets

The produced tablets were evaluated in terms of quality attributes. The weight of the X2T (xylitol-based tablet) was in the 599–602 mg range with deviation from the mean being less than 1%. Similarly, the weight of M2T (menthol-based tablet) ranged from 633 to 637 mg with a % deviation less than 1% which complies with the acceptable range of ± 5%. This was reflected in the thickness of the tablets which was 3.64 ± 0.075 and 4.14 ± 0.074 for X2T and M2T, respectively. Drug content ranged from 92 to 102% for the X2T tablet and between 100.5 to 109% for the M2T tablet of the potency indicating homogeneity of mixing. The tablet hardness was 4.4 ± 0.14 and 4.65 ± 0.14 Kp for X2T and M2T, respectively. The computed friability values were 0.52% for X2T and 0.89% for M2T tablets. The disintegration time was 25 and 30 s for X2T and M2T tablets, respectively. The fast disintegration characteristics were reflected further by the short wetting time values which were 20.7 ± 1.15 s for X2T tablets and 23 ± 1.7 s for M2T tablets.

**Figure 5:**
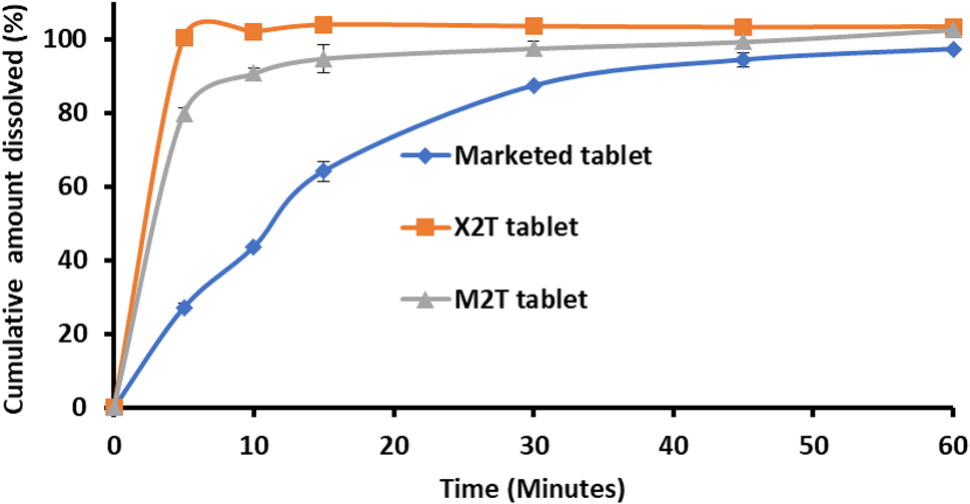
In vitro dissolution of bosentan from the developed orodispersible tablets and the marketed tablets. Formulation details are in Table 2.

Table 6 presents the dissolution parameters and Fig. 5 shows the dissolution profile of bosentan from (X2T), (M2T), and the marketed tablets. Xylitol-based tablets underwent ultrafast dissolution as reflected by almost complete dissolution after 5 min with an overall dissolution efficiency of 98.7%. This dissolution behavior is comparable to that recorded from the corresponding formulation. This indicates the absence of a negative effect on the dissolution characteristics of xylitol-based formulation after compaction. For menthol-based tablets (M2T), the tablet liberated 79.9% of bosentan within the first five minutes with a 92.01 dissolution efficiency (Table 6 and Fig. 5). These values are relatively lower than the corresponding formulation. The marketed product underwent slow dissolution in the first 10 min as indicated by the Q5 and Q10 values which recorded 27.14% and 43.7%, respectively. This tablet liberated 87.2% after 30 min. Statistical comparison reflected the superiority of the developed oral disintegrating tablets (X2T and M2T) over the marketed product with respect to dissolution parameters (F2 = 7 and 16 for X2T and M2T, respectively).

**Table 6:** The dissolution parameters of the prepared tablets after storage.

Marketed tablet						
Q5 (%)	Q10 (%)	Q60 (%)	DE (%)			
27.14 ± 1.11	43.71 ± 0.51	97.18 ± 0.48	68.62 ± 0.90			
X2T						
Time (months)	Q5 (%)	Q10 (%)	Q60 (%)	DE (%)	DT (seconds)	F2 values relative to marketed product
0	100.37 ± 0.31	101.91 ± 0.55	103.33 ± 1.05	98.7 ± 0.32	30	7
1	96.64 ± 1.60	99.52 ± 1.25	108.51 ± 1.47	99.7 ± 0.94	27	8
2	97.32 ± 1.03	102.0 ± 0.73	104.18 ± 0.43	99.03 ± 0.61	28	8
3	97.08 ± 0.84	102.28 ± 0.86	104.44 ± 0.08	98.7 ± 0.72	30	8
M2T						
0	79.96 ± 1.41	90.69 ± 1.46	102.5 ± 1.56	92.01 ± 1.90	25	16
1	79.63 ± 1.11	88.05 ± 1.89	100.36 ± 0.50	91.5 ± 1.33	25	16
2	80.31 ± 1.42	91.32 ± 1.05	101.28 ± 1.41	93.06 ± 0.92	27	15
3	83.04 ± 0.90	90.33 ± 2.78	95.3 ± 0.53	90.08 ± 1.24	29	14

The similarity factor test of each tablet dissolution at different time intervals showed the absence of any change upon storage (F2 > 50% in all cases).

### Stability studies

The stability of the developed tablets was investigated by monitoring the disintegration time and dissolution characteristics after storage at ambient temperature. These parameters were selected for stability studies as they are considered the most critical criteria for rapidly disintegrating formulations. Table 6 lists the results. The outcomes made sure that the crucial characteristics of rapidly disintegrating tablets would be preserved with following fast dissolution throughout the study period. This was demonstrated by the fact that the disintegration time and dissolving properties of tablets with storage had no appreciable change. This was supported by statistical analysis (*P* > 0.05, Table 6). The similarity factor test confirmed further no significant change in the dissolution characteristics upon storage.

## Discussion

FTIR spectral analysis of pure bosentan was parallel to its chemical function groups which were similarly assigned in the corresponding literature^1,21,40,41^. Similarly, the spectral pattern of xylitol complied with its chemical structure and the recorded spectrum is similar to the published spectrum for unprocessed xylitol^26^. Being terpene alcohol, the FTIR spectrum of menthol showed a broad absorption band for the OH stretching as the main absorption band with the spectrum showing the rest of the functional groups accounting for its structure. Similar results were found by other researchers^34,42^.

Wet co-grinding of bosentan with xylitol yielded a product with FTIR spectrum which differs from the combination of the ingredients of the mixture. The modifications depended on the bosentan-to-xylitol ratios with distinct differences being shown at higher concentrations of xylitol. The modulations included broadening and/or shifting of the absorption bands corresponding to hydrogen bonding sites. These findings reflect at least hydrogen bonding between xylitol and bosentan which is acceptable taking into consideration the existence of hydrogen bonding sites in both compounds. A similar supposition was postulated after recording alike changes after the co-processing of other compounds^26^.

Similarly, the co-processing of bosentan and menthol produced spectral changes highlighting possible hydrogen bonding. Noteworthy, the magnitude of change in the spectrum after co-processing with menthol is lower than that recorded after kneading with xylitol. This is obvious taking into consideration the existence of only one hydrogen-bonding site in the case of menthol as compared with the multiple sites in the case of xylitol (Fig. 1).

The thermogram of the raw material of bosentan produced three successive thermal events, the first endotherm indicates the liberation of water of crystallization and the second denotes the melting of bosentan crystals. The late exotherm indicates the thermal events for bosentan decomposition. Similar thermal behavior has been reported by other authors who suggested similar interpretations^1,43^. The thermal pattern of raw xylitol showed melting and decomposition endothermic transitions. This data correlates with that reported in literature reports^26^. For menthol, the thermal pattern of menthol exposed sharp and broad endothermic peaks reflecting its melting and decomposition, respectively. The same pattern was seen by other researchers^34,44^.

Ethanol influenced kneading of bosentan with xylitol produced a solid product with thermal behavior showing modulation in the melting transition of bosentan compared with the untreated material. The modulations suggest at least partial amorphization with possible size reduction. A Similar explanation was reported in other studies after recording a reduction in the enthalpy of melting transition after processing^28,45^.

The kneading of bosentan with menthol produced a dry product with modulated thermograms relative to pure bosentan. These alterations suggest eutectic mixture formation and/or partial amorphization. The extent of modulation increased with increasing molar ratio of menthol reflecting the need of menthol at high concentration. Similar discussions were reported by other researchers after recording similar alterations in the thermal behavior of the drug following treatment with formulation additives^45^.

The X-ray diffraction of pure bosentan demonstrated its crystalline structure and showed similar diffraction patterns to that reported by other researchers^1,46^. Similarly, the crystalline structure of xylitol was shown from its diffractogram which resembles that of xylitol from previous studies^26^. The Diffractogram of pure menthol was similar to that reported for the same crystalline material^34^. Broadening and reduction in the intensity of diffraction peaks after kneading with xylitol support partial amorphization along with reduced particle size. A Similar supposition was recorded by other research teams after noting equivalent changes^46^.

Kneading with menthol produced solid crystals with an XRD pattern supporting partial amorphization, eutexia with size reduction as indicated from the preservation of the spacing of diffraction peaks, broadening, and lower intensity. A similar supposition was recorded by other research teams after noting equivalent changes in X-ray diffraction patterns along with thermal analysis and FTIR spectroscopy^27^.

Monitoring of Bosentan dissolution was done using its untreated powder and mixtures that have been coprocessed with xylitol or menthol. The slow dissolution of unprocessed bosentan is expected considering the physicochemical characteristics of the drug and is comparable to that reported in literature studies on bosentan dissolution^16–21,46^. Kneading of bosentan with ethanol in the absence of any additives did not alter the dissolution significantly compared with the unprocessed bosentan. This indicates that ethanol-aided kneading of bosentan without excipient did not alter its physical properties and any change after co-processing with additives will result from the effect of the tested additive. The enhanced dissolution rate of bosentan when co-processed with xylitol can be justified based on the recorded partial amorphization after ethanol-assisted kneading. Partial amorphization can weaken the intermolecular bonds allowing faster bosentan dissolution. A contribution from the solubilizing or hydrotropic effects of xylitol on hastened dissolution was shown by the recorded increase in bosentan dissolution from simple physical mixing compared to net bosentan. However, crystalline structure modification is the main contributor to dissolution enhancement as indicated by the superiority of formulation over physical mixture. The superiority of formulation containing higher proportion of xylitol is correlated with the recorded higher extent of modulation in FTIR spectral pattern and thermal behavior. A Similar change was shown to increase the release rate of some drugs like itraconazole, tadalafil, bicalutamide, and carvedilol^28,45,47,48^.

Menthol induced dissolution improvement can be attributed to melting point reduction which is alleged to reduce intermolecular forces providing faster dissolution^27,31–33^. Menthol was reported to augment the dissolution rate of lopinavir, but the reason for this effect was certified to the development of co-crystals^34^. Menthol was also involved in the formulation of simple solid dispersion to hasten the solubility and the dissolution rate of sulfamethoxazole^42^. As for xylitol, physical mixing with menthol showed some degree of dissolution increase relative to pure drug. This suggests a solubilizing or hydrotropic effect of menthol. However, this suggestion requires further investigation in the light of poor water solubility of menthol.

The adequacy of the flow behaviors of the powdered mixture of tablet formulation was ensured from the results of the Carr index, Hausner`s ratio, and angle of repose. This flow behavior was further reflected in the characteristics of the prepared tablets which were of uniform weight and drug content. The cohesiveness of the powder was shown from the hardness and friability values which were acceptable for oral disintegrating tablets^36,49^. The fast disintegration time satisfies the FDA specification of orodispersible tablets which recommended tablet disintegration in less than or equal to 30 s^28^. Parallel with that ultrashort wetting time was noticed. The ultrafast disintegration and short wetting time can reflect the porous nature of the prepared tablets which are imparted from crospovidone and croscarmellose which are categorized as super disintegrants. These materials undergo rapid absorption of the aqueous media, swelling with subsequent disintegration^28,49^.

The dissolution parameters of tablets were relatively lower than the corresponding powdered formulation. This imitates possible size enlargement after compaction which is expected for eutectic forming systems due to melting point depression and cohesive/adhesive forces. Despite this finding, the developed tablet underwent a dissolution pattern acceptable for rapidly dissolving tablets. The recorded initial slow dissolution from marketed tablets is acceptable taking into consideration the fact that the marketed tablet is categorized as an immediate release tablet and not as a rapidly disintegrating orodispersible tablet. The recorded fast dissolution of the developed tablets correlates with the short wetting time and ultrashort disintegration time. The development of rapidly disintegrating tablets has been documented to accelerate the dissolving rate of a variety of hydrophobic drugs^28,49–51^.

Along with enhanced dissolution, the developed tablets retained their principal characteristics even after storage for 3 months suggesting the compatibility of selected excipients, but this requires verification by full term stability study.

## Conclusion

Wet co-processing of bosentan with xylitol or menthol modified the crystalline structure. The modification was in the form of partial amorphization in the case of xylitol with menthol inducing melting point depression. These modifications hastened the dissolution rate of bosentan and the product was easily fabricated as a rapidly disintegrating tablet for intra-oral administration. The developed tablets showed ultrafast disintegration with the subsequent release of most of the dose. The study thus developed a children and geriatric friendly tablet of bosentan using simple co-processing with benign excipients. However, the palatability study is recommended for future optimization.

## Data Availability

The datasets generated and/or analyzed during the current study are available from the corresponding author upon reasonable request.
